# The Contribution of Diet Quality to Socioeconomic Inequalities in Obesity: A Population-based Study of Swiss Adults

**DOI:** 10.3390/nu11071573

**Published:** 2019-07-12

**Authors:** Carlos de Mestral, Angeline Chatelan, Pedro Marques-Vidal, Silvia Stringhini, Murielle Bochud

**Affiliations:** 1 Department of Epidemiology and Health Systems, Center for Primary Care and Public Health (Unisanté), University of Lausanne, Route de la Corniche 10, 1010 Lausanne, Switzerland; 2 Unit of Population Epidemiology, Division of Primary Care Medicine, Department of Community Medicine, Primary Care and Emergency Medicine, Geneva University Hospitals, Rue Gabrielle-Perret-Gentil 4, 1205 Geneva, Switzerland; 3 Department of Internal Medicine, Internal Medicine, Lausanne University Hospital, Rue du Bugnon 46, 1005 Lausanne, Switzerland

**Keywords:** diet quality, socioeconomic status, inequalities, education, income, obesity, 24 h dietary recall

## Abstract

Socioeconomically disadvantaged people are disproportionally more likely to develop obesity and obesity-related diseases. However, it remains unclear to what extent diet quality contributes to socioeconomic inequalities in obesity. We aimed to assess the role of diet quality in the association between socioeconomic status (SES) and obesity. Data originated from the national nutrition survey, a cross-sectional sample of the adult Swiss population (*N* = 1860). We used education and income as proxies for SES; calculated the Alternate Healthy Eating Index (AHEI) as a measure of diet quality; and used body mass index (BMI), waist circumference (WC), waist-to-hip ratio (WHR), and waist-to-height ratio (WHtR) as obesity markers. We applied counterfactual mediation modelling to generate odds ratios, 95% confidence intervals, and the proportion mediated by diet quality. Individuals with less than a tertiary education were two to three times more likely to be obese, regardless of the marker (OR (95% CI): 3.36 (2.01, 5.66) using BMI; 2.44 (1.58, 3.75) using WC; 2.48 (1.63, 3.78) using WHR; and 2.04 (1.43, 2.96) using WHtR). The proportion of the association between educational level and obesity that was mediated by diet quality was 22.1% using BMI, 26.6% using WC, 31.4% using WHtR, and 35.8% using WHR. Similar findings were observed for income. Our findings suggest that diet quality substantially contributes to socioeconomic inequalities in obesity while it does not fully explain them. Focusing efforts on improving the diet quality of disadvantaged groups could help reduce social inequalities in obesity.

## 1. Introduction

Obesity is a main risk factor for the development of type 2 diabetes, hypertension, cardiovascular disease, and cancer [1,2]. Its prevalence follows a social gradient in populations of high-income countries (HICs), whereby socioeconomically disadvantaged people are much more likely to be obese compared with people who are more privileged. This social gradient in obesity is observed across several adiposity markers, including body mass index (BMI) [3,4], waist circumference (WC) [5,6,7], and waist-to-hip ratio (WHR) [8,9]. Socioeconomically disadvantaged people face thus a greater risk of developing obesity-related chronic conditions and of dying prematurely [10,11]. In Switzerland—a wealthy country whose population exhibits one of the highest life expectancies and among the lowest prevalence rates of chronic disease risk factors worldwide—the prevalence of obesity (body mass index ≥ 30 kg/m^2^) is approximately 5% among individuals with higher levels of education, and three times higher (15%) among individuals with lower levels of education [12,13]. It is also well established that unhealthy eating leads to weight gain [14,15], and that individuals in socioeconomically disadvantaged groups are more likely to follow nutrient-poor and energy-dense diets—consuming fewer fruits, vegetables, legumes, whole grains, nuts, seeds, lean meats, and fish, but consuming more highly processed foods and red meats, refined grains, and sugar-sweetened beverages [16,17,18]. It is therefore likely that diet quality contributes to the widespread socioeconomic inequalities in obesity rates.

To date, few studies have examined the contribution of diet quality in the association between socioeconomic status (SES) and obesity. These studies have found that diet contributed to between 12% and 50% of the socioeconomic inequalities in obesity [19,20,21,22,23]. Importantly, these studies presented limitations in their findings. Firstly, they relied on self-reported measures and predominantly used BMI to define obesity, which only reflects total body fat and not visceral fat [24]. In population-based surveys, reliable estimates of visceral fat include WC, WHR, and waist-to-height ratio (WHtR) [24,25,26]. Other measures of body fat such as bioimpedance [27] or dual-energy X-ray absorptiometry (DEXA) are less used as they depend on the method used (bioimpedance) [28] or are too expensive to apply (DEXA) in epidemiological studies. Secondly, and crucially, these studies did not assess diet quality comprehensively—mostly focusing on single food groups [19,20,21,22,23]. We thus aimed to assess the contribution of diet in the association between education and obesity in Switzerland using a comprehensive measure of diet quality, the Alternate Healthy Eating Index (AHEI). We hypothesized that diet quality would substantially contribute to the socioeconomic inequalities in obesity.

## 2. Materials and Methods

We followed the STROBE recommendations for reporting. We used data from the population-based cross-sectional survey menuCH, conducted in 2014–2015 among non-institutionalized adults aged 18 to 75 years in the Swiss population. The study was approved by all regional ethics committees and adheres to the Declaration of Helsinki principles. Trained dietitians assessed dietary intake via two non-consecutive multi-pass computer-assisted 24-hour dietary recalls [29]. The menuCH study sampling and methodology is described in detail elsewhere [29]. 

### 2.1. Socioeconomic Status

Education was dichotomized into (1) tertiary education and (2) secondary/primary education (see Table S1 for detailed description of educational level). Monthly household income was categorized into three groups: (1) ≤ 6000 CHF, (2) >6000 to <9000 CHF, and (3) ≥ 9000 CHF (1.00 CHF = 1.00 USD = 0.90 EUR).

### 2.2. Diet Quality

We estimated diet quality via the 2010 version of the Alternate Healthy Eating Index (AHEI), which has been extensively used to study the association between diet quality and a series of chronic diseases, as well as cause-specific and all-cause mortality [30,31]. Briefly, the AHEI is an index composed of 11 food- and nutrient-specific components (Table S2), including vegetables, fruit, whole grains (defined as a carbohydrates-to-fiber ratio ≤ 10:1), sweetened beverages and fruit juices, nuts and legumes, red and processed meats, trans-fat, fish (as a proxy for long-chain n-3 fatty acids), polyunsaturated fatty acids, sodium, and alcoholic drinks intake [31]. The total score ranges from 0 (worst diet quality) to 110 (optimal diet quality). We created quintiles of the AHEI to use in the analysis, with the lowest quintile representing the unhealthiest diet quality and the highest quintile the healthiest diet quality. For sensitivity analyses, we computed the Mediterranean Diet Score (MDS), another comprehensive measure of diet quality extensively shown to be associated with reduced total mortality [32,33]. This score awards 1 point for an intake equal to or above the sex-specific median for vegetables, legumes, fruit and nuts, cereal products, fish, and ratio of monounsaturated to saturated fat, and 1 point for an intake below the sex-specific median for harmful foods (i.e., meat and dairy products). Moderate alcohol intake was awarded by 1 point if consumption was between 5 and 25 g/day for women, and 10 and 50 g/day for men (Table S3). The total score ranged from 0 to 9 points, with a higher score corresponding to higher adherence to the Mediterranean diet [34].

### 2.3. Obesity Markers

We used four different measures of obesity, all of which have been shown to be associated with adiposity, risk of type 2 diabetes [35], and cardiovascular disease [36,37]. The following obesity markers were calculated from height, weight, and waist and hip circumference objectively measured by trained dietitians: (1) body mass index (BMI), (2) waist circumference (WC), (3) waist-to-hip ratio (WHR), and (4) waist-to-height ratio (WHtR). For each obesity marker, we defined obesity as follows: (1) BMI ≥ 30 kg/m^2^; (2) WC > 102 cm for men, > 88 cm for women; (3) WHR ≥ 0.90 for men, ≥ 0.85 for women; and (4) WHtR ≥ 0.5 for both men and women as recommended [25,38].

### 2.4. Covariates

We used age (continuous), sex, total energy intake (daily kilocalories), self-reported smoking (smokers versus non-smokers), and physical activity. Physical activity was assessed with the short-form International Physical Activity Questionnaire (IPAQ, six questions); data were converted into Metabolic Equivalent of Task (MET) minutes per week [39] and categorized according to IPAQ guidelines into low, moderate, and high physical activity levels.

### 2.5. Statistical Analysis

We excluded from the analysis participants below the age of 25 years (as these may be pursuing an education), those without two valid 24 h dietary recalls, and those lacking data on educational level or obesity markers. We imputed missing values of the six IPAQ questions (between 1 and 16% of missing value for a single question) to calculate MET-min per week using chained multiple imputations (20 imputations) by predictive mean matching through a Markov chain Monte Carlo method. After examining whether socioeconomic inequalities in obesity differed by gender, and finding that they did not (Table S4), we chose to combine men and women in the analyses. We assessed the association between education and quintiles of the AHEI using ordered logistic regression, adjusting for age, sex, and physical activity. Then, we assessed the association between quintiles of the AHEI and each of the obesity markers, adjusting for age, sex, physical activity, and total energy intake. We tested for linear trends across quintiles of the AHEI. Finally, we examined the mediation of diet quality in the association between education and obesity markers using the counterfactual method of mediation analysis [40,41], adjusting for age, sex, physical activity, total energy intake, and smoking behavior. This method calculates the following components: (1) the natural direct effect (NDE, in Odds ratio), the effect of the exposure (education) on the outcome (obesity) via pathways that exclude the mediator (diet quality); (2) the natural indirect effect (NIE), the effect of the exposure on the outcome via the mediator; (3) the marginal total effect (MTE), the total effect of the exposure on the outcome; and (4) the proportion mediated (PM), the proportion of the exposure–outcome association that is mediated by the mediator. We computed 95% confidence intervals for the above counterfactual components using bootstrap procedures with 1000 simulations [40]. In sensitivity analyses, we repeated the analyses in four ways: (1) using the difference method to assess mediation, (2) using the MDS as the measure of diet quality, (3) using a three-level educational level exposure (higher tertiary; lower tertiary and higher secondary; lower secondary and primary education), and (4) using income instead of education as the SES measure. All analyses were conducted using Stata version 15 (StataCorp, College Station, TX, USA).

### 2.6. Ethics

This survey followed the guidelines set in the Declaration of Helsinki and all procedures were approved by the corresponding regional ethics committees (lead committee in Lausanne, Protocol 26/13, approved on 12 February 2013). Written informed consent was obtained from all participants. The survey was registered (International Standard Randomized Controlled Trial Number (ISRCTN): ISRCTN16778734).

## 3. Results

### 3.1. Characteristics of the Sample

Table 1 shows the characteristics of the included sample, overall and by educational level. In total, 1860 participants were included in the analysis (see Figure S1 for participant inclusion), of which 54% were women, with a mean age of 49 years. Participants with a tertiary education (52%) were younger than participants with lower education (mean age 47 y versus 52 y, respectively), and there were more women in the lower education group (61% versus 48%). The mean AHEI was 48.8, higher among participants with a tertiary education (Table S2, for score by component). The prevalence of obesity ranged from 11% for BMI-derived obesity to 43% for WHtR-derived obesity, with marked differences between the educational groups irrespective of the marker.

### 3.2. Associations between Education, Dietary Quality and Obesity

Table 2 displays the age, sex, and physical activity-adjusted association between educational level and quintiles of the AHEI. A clear gradient emerged showing that having less than a tertiary education was associated with a worse diet. Compared with participants having a tertiary education, those with lower than a tertiary education were 1.60 times more likely (95% CI: 1.18, 2.15) to have their diet quality in the unhealthier quintile (mean, SD: 39.5, 2.3), and 2.29 (1.68, 3.12) times more likely to be in the unhealthiest quintile (mean, SD: 29.1, 4.6).

Table 3 reveals the association between quintiles of the AHEI and each obesity marker. A clear gradient became evident in this association as well—the poorer the diet quality, the higher the likelihood of obesity, irrespective of the marker. Participants having the worst diet quality were more than three times more likely to be obese, compared with participants having the best diet quality.

### 3.3. Mediation of Diet Quality in the Education-obesity Association

Table 4 shows results of the counterfactual mediation analysis. These indicated that having less than a tertiary education was associated with higher likelihood of obesity, regardless of the obesity marker. The marginal total effect of having less than a tertiary education on obesity ranged from 2.04 (1.43, 2.96) for WHtR-derived obesity to 3.36 (2.01, 5.66) for BMI-derived obesity. Diet quality contributed to 22.1% of the inequalities in obesity using BMI, to 26.6% using WC, to 31.4% using WHtR, and to 35.8% using WHR as the obesity marker.

In sensitivity analyses, applying the difference method to assess mediation revealed that diet quality explained a lower proportion of the association between education and obesity (Table S5). Categorizing educational level into three groups yielded similar results, albeit larger effect sizes and wider confidence intervals (Tables S6 and S7). Using the MDS instead of the AHEI indicated that diet quality explained between 9% and 14% of the association between education and obesity (Table S8). Finally, using income as the measure of SES yielded findings that reflected those using educational level (Tables S9–S11).

## 4. Discussion

In this sample of adults in the Swiss population, we found that irrespective of the obesity marker used, participants with a secondary/primary education were more likely to be obese than participants with a tertiary education—findings that reflect those in the literature [10,11,12,13,14]. Participants with less than a tertiary education were also more likely to have diets of poorer quality; in turn, a poorer diet quality was associated with obesity, regardless of the obesity marker. These findings also accord with published reports [15,16,17,18]. Consequently, as we had hypothesized, we found evidence of substantial mediation by diet quality in the association between SES and obesity.

Comparing our mediation results to the literature is limited by the fact that previous reports have used widely differing analytical mediation methods [19,20,21,22,23], relied on self-reported measures of obesity (mostly BMI-derived obesity) [20,22,23], and most importantly, assessed diet quality in a less comprehensive way [19,20,21,22,23]. For instance, in an Australian sample of 840 participants, an “unhealthy take-away foods” index explained 15% of educational inequalities in BMI, although a “healthy take-away foods” index showed no mediation [22]. Similarly, in a sample of 6037 adults from five European urban areas, vegetables intake explained 8% of the association between neighborhood socioeconomic status and BMI, while fruit, soft drinks, and sweets intake failed to explain any part of the association [20]. Despite methodological differences, our findings are in line with these reports and provide a more precise, accurate, and comprehensive estimation of the contribution of overall diet quality to socioeconomic inequalities in obesity.

### 4.1. Implications for Public Health

Given the continuous expansion of obesity and its deleterious effects on chronic disease risk and quality of life, our findings strengthen the argument to strive for improvements in diet quality among disadvantaged populations. To achieve this, two complimentary steps are needed. The first step consists of major structural changes to transform the current food system and food environment into ones that facilitate and promote healthy eating behaviors, which would drive a population-wide shift towards healthier diets, as public health experts have extensively argued [14,15,42,43,44]. The second, and crucial, step consists of targeted interventions that address the specific circumstances, needs, and challenges of socioeconomically disadvantaged groups that are less likely to benefit from population-wide improvements in the food environment [45]. Together with improvements of socioeconomic conditions in general, this approach may, in the context of our study, enable individuals with less than a tertiary education, or with lower income, to achieve and maintain healthier diets that are more similar to those of individuals with a tertiary education or higher income. This may, in turn, mitigate socioeconomic inequalities in obesity and health as well as reduce the overall disease burden in the population. To date, however, most interventions to improve healthy eating in the general population and in specific groups have relied on individual responsibility, primarily in relation to nutrition knowledge [42,46,47], even though the evidence shows these have limited impact and often exacerbate inequalities [14,47,48,49].

### 4.2. Study Strengths and Limitations

A main strength of our sample lies in the comprehensive dietary intake measurements using two 24h dietary recalls, and the use of objectively measured markers of obesity. To our knowledge, ours is the first study to assess the mediation of diet quality using validated and comprehensive measures of diet—the AHEI and MDS—calculated from dietary intake based on two 24h dietary recalls, and using objective markers of obesity. Additionally, the counterfactual method provides a more accurate assessment of mediation in the presence of interaction between the exposure and the mediator [40,41]. As shown in the sensitivity analysis, the difference method found reduced mediation of diet as it fails to account for potential diet–SES interaction. Finally, our findings were similar when using two different measures of SES—education and income.

The main limitation of our study is the cross-sectional nature of the data, which does not allow for temporal examination of exposure, mediator, and outcome. The sample size is also suboptimal, which likely explains the broad confidence intervals. It is likely that participants forgot reporting some consumed foods (recall bias) or underreported their intake of well-known unhealthier foods and over-reported their intakes of healthier foods (social desirability bias). Participants with fewer years of education are more likely to underreport food intake [50], although this would have biased the observed associations towards the null. Nevertheless, the consistent associations we found in our analysis (between exposure and mediator, mediator and outcome, and exposure and outcome) are in agreement with an extensive literature linking diet quality to obesity and socioeconomic conditions. Participants in the survey may have been more health- and nutrition-conscious than non-participants. However, the prevalence of BMI obesity in our sample was similar to the one reported in other Swiss surveys with higher response rates [12,51,52].

## 5. Conclusions

Our findings suggest that diet quality substantially contributes to socioeconomic inequalities in obesity in this sample of the adult Swiss population. Improving the diet quality of socioeconomically individuals to resemble that of more advantaged individuals could potentially substantially reduce inequalities in obesity and obesity-related conditions.

## Figures and Tables

**Table 1:** Description of the included sample, menuCH survey, Switzerland, 2014–2015.

	Total	Educational Level		*p*-Value
		Tertiary	Secondary/Primary	
N	1860	972 (52.3)	888 (47.7)	
Women, n (%)	1009 (54.2)	469 (48.3)	540 (60.8)	<0.001
Age, mean (SD)	49.2 (14.1)	46.8 (14.0)	51.9 (13.7)	<0.001
Smoking, n (%)	401 (21.6)	209 (21.5)	192 (21.6)	0.95
Physical activity				<0.001
Low	110 (5.9)	60 (6.2)	50 (5.6)	
Moderate	807 (43.4)	509 (52.4)	298 (33.6)	
High	943 (50.7)	403 (41.5)	540 (60.8)	
AHEI, mean (SD)	48.8 (14.3)	49.6 (14.3)	47.8 (14.3)	0.01
AHEI quintiles, median (range)				
Healthiest	68.6 (60.6–97.3)	69.3 (60.6–97.3)	68.2 (60.7–94.0)	0.26
Healthier	55.6 (51.2–60.6)	55.7 (51.3–60.5)	55.2 (51.2–60.6)	0.61
Middle	47.3 (43.2–51.2)	47.5 (43.2–51.1)	47.3 (43.2–51.2)	0.97
Unhealthier	39.5 (35.2–43.1)	39.3 (35.2–43.0)	39.5 (35.3–43.1)	0.91
Unhealthiest	29.4 (12.4–35.2)	29.9 (16.0–35.2)	29.3 (12.4–35.2)	0.72
Obese, n (%) by marker				
Body mass index	196 (10.6)	70 (7.2)	126 (14.2)	<0.001
Waist circumference	344 (18.8)	128 (13.4)	216 (24.6)	<0.001
Waist-to-hip ratio	549 (30.0)	252 (26.4)	297 (33.8)	0.001
Waist-to-height ratio	787 (43.0)	344 (36.0)	443 (50.5)	<0.001

AHEI, Alternate Healthy Eating Index. Statistical differences between educational groups for categorical variables assessed by Chi-square test, for age and the AHEI using student T-test; differences in medians between educational groups within each quintile assessed with Wilcoxon rank-sum test.

**Table 2:** Association of educational level with quintiles of the Alternate Healthy Eating Index, menuCH, Switzerland, 2014–2015.

AHEI Quintiles	Mean (SD)	Primary/Secondary vs. Tertiary
		OR (95% CI)
Healthiest	69.5 (7.1)	1.00 (reference)
Healthier	55.6 (2.8)	1.16 (0.86, 1.56)
Middle	47.2 (2.3)	1.34 (1.00, 1.80)
Unhealthier	39.5 (2.3)	1.60 (1.18, 2.15)
Unhealthiest	29.1 (4.6)	2.29 (1.68, 3.12)
P-trend ^a^		<0.001

AHEI, Alternate Healthy Eating Index; OR, odds ratio; CI, confidence interval. Odds ratio and 95% confidence interval adjusted for age, sex and physical activity, from ordered logistic regression, comparing likelihood of being in each quintile of the AHEI for less than university education relative to university education. ^a^ *P*-value for linear trend.

**Table 3:** Association between quintiles of the Alternate Healthy Eating Index and obesity markers, menuCH, Switzerland, 2014–2015.

Obesity Marker	AHEI Quintiles					P-Trend ^a^
	Healthiest	Healthier	Middle	Unhealthier	Unhealthiest	
	Reference	OR (95% CI)	OR (95% CI)	OR (95% CI)	OR (95% CI)	
Body mass index	1.00	1.22 (0.70, 2.12)	1.91 (1.14, 3.20)	2.09 (1.25, 3.49)	3.42 (2.06, 5.67)	<0.0001
Waist circumference	1.00	1.58 (1.05, 2.38)	1.76 (1.18, 2.65)	2.32 (1.55, 3.47)	3.42 (2.26, 5.17)	<0.0001
Waist-to-hip ratio	1.00	1.72 (1.17, 2.53)	1.89 (1.29, 2.78)	2.66 (1.81, 3.90)	3.30 (2.23, 4.91)	<0.0001
Waist-to-height ratio	1.00	1.82 (1.30, 2.54)	2.16 (1.54, 3.02)	3.11 (2.20, 4.38)	3.89 (2.72, 5.56)	<0.0001

AHEI, Alternate Healthy Eating Index; OR, odds ratio; CI, confidence interval. Odds ratio and 95% confidence interval for the likelihood of being in obesity category according to each obesity marker, for individuals in each quintile of the AHEI relative to those in the highest (healthiest) quintile (reference group), adjusted for age, sex, physical activity, and total energy intake. ^a^ Trend across quintiles of AHEI.

**Table 4:** Counterfactual mediation of diet quality (AHEI) in the association of educational level with obesity markers, menuCH, Switzerland, 2014–2015.

Obesity Marker	MTE	NDE	NIE	PM
	OR (95% CI)	OR (95% CI)	OR (95% CI)	% (95% CI)
Body mass index	3.36 (2.01, 5.66)	2.84 (1.68, 4.94)	1.18 (1.06, 1.36)	22.1 (8.5, 41.6)
Waist circumference	2.44 (1.58, 3.75)	2.06 (1.34, 3.18)	1.19 (1.07, 1.34)	26.6 (11.4, 48.4)
Waist-to-hip ratio	2.48 (1.63, 3.78)	2.01 (1.35, 3.03)	1.23 (1.10, 1.41)	31.4 (16.1, 53.3)
Waist-to-height ratio	2.04 (1.43, 2.96)	1.67 (1.18, 2.39)	1.22 (1.11, 1.39)	35.8 (19.2, 63.4)

AHEI, Alternate Healthy Eating Index; MTE, marginal total effects; NDE, natural direct effect; NIE, natural indirect effect; OR, odds ratio; CI, confidence interval. Odds ratio and 95% confidence interval for the total effect of the exposure on the outcome (MTE), for the effect of the exposure on the outcome via pathways that exclude the mediator (NDE); the effect of the exposure on the outcome via the mediator (NIE). PM, proportion of the association between educational level and obesity markers which is mediated by diet quality, estimated using the AHEI, adjusted for age, sex, physical activity, total energy intake, and smoking behavior.

## Supplementary Materials

The following are available online at https://www.mdpi.com/2072-6643/11/7/1573/s1, Table S1: Definition and categorization of educational level, menuCH, Switzerland, 2014–2015; Table S2: Alternate Healthy Eating Index components and scoring^a^, menuCH, Switzerland, 2014–2015; Table S3: Mediterranean diet score (MDS) components and scoring, menuCH, Switzerland, 2014–2015; Table S4: Description of included sample, menuCH, Switzerland, 2014–2015; Table S5: Association of educational level with obesity outcomes, and the mediation of diet (AHEI) in this association, assessed via the difference method, menuCH, Switzerland, 2014–2015; Table S6: Association between educational level^a^ and quintiles of the Alternate Healthy Eating Index, menuCH, Switzerland, 2014–2015; Table S7: Results from counterfactual mediation of diet quality (AHEI) in the association of educational level^a^ with obesity markers, menuCH, Switzerland, 2014–2015; Table S8: Results from counterfactual mediation of diet quality (MDS) in the association of educational level with obesity markers, menuCH, Switzerland, 2014–2015; Table S9: Association between income level and quintiles of the Alternate Healthy Eating Index, menuCH, Switzerland, 2014–2015; Table S10: Association between quintiles of the Alternate Healthy Eating Index and obesity markers, menuCH, Switzerland, 2014–2015; Table S11: Counterfactual mediation of diet quality (AHEI) in the association of income level with obesity markers, menuCH, Switzerland, 2014–2015; Figure S1: Flowchart of participant inclusion, menuCH, Switzerland, 2014–2015.
