# Supplementary material for: The Contribution of Diet Quality to Socioeconomic Inequalities in Obesity: A Population-based Study of Swiss Adults
Source: Nutrients. 2019 Jul 12;11(7):1573. doi: 10.3390/nu11071573 (PMC6683031; doi:10.3390/nu11071573)

Supplementary material to

The contribution of diet quality to socioeconomic inequalities in obesity: A population-based study of Swiss adults

Table S1. Definition and categorization of educational level, menuCH, Switzerland, 2014-2015

Educational level	Definition
Primary	Primary school (compulsory school):<br>- Eight years of schooling, from age 4-6 onwards
Lower secondary	Vocational training (1-4 years after compulsory school)<br>- Pre-apprenticeship, vocational internship, one-year vocational school<br>- Apprenticeship, vocational training
Higher secondary	High school (3-4 years after compulsory school)<br>- Specialized professional or vocational school<br>- High school
Lower tertiary	Higher vocational training (5-7 years after compulsory school)<br>- Higher vocational school with specialization<br>- Technical vocational training with specialization years)
Higher tertiary	University (> 6-7 years after compulsory school)<br>- Bachelor degree<br>- Master degree<br>- Doctoral degree

Further information regarding the Swiss educational system:
https://www.swissuniversities.ch/en/higher-education-area/swiss-education-system/

Table S2. Alternate healthy eating index components and scoring[a], menuCH, Switzerland, 2014-2015

Component	Criteria for minimum score of 0	Criteria for maximum score of 10	Tertiary education (N = 972)	Lower education (N = 888)
Vegetables (servings/day)[b]	0	≥ 5	3.5 ± 2.1	3.2 ± 2.0
Fruit (servings/day)[c]	0	≥ 4	3.8 ± 3.2	3.5 ± 3.1
Whole grains (g/day)[d]			4.0 ± 3.9	3.4 ± 3.8
Women	0	≥ 75		
Men	0	≥ 90		
Sugar-sweetened beverage and fruit juice (servings/day)[e]	≥ 1	0	5.0 ± 4.2	5.4 ± 4.5
Nuts, seeds, legumes, and tofu (servings/day)[f]	0	≥ 1	2.3 ± 3.6	1.8 ± 3.3
Red and processed meat (servings/day)[g]	≥ 1.5	0	4.2 ± 3.9	4.0 ± 4.0
Trans fatty acids (% of total energy intake)[h]	≥ 4	≤ 0.5	9.4 ± 0.3	9.4 ± 0.3
Fish, excluding processed products (g/day)[i]	0	32.4	2.8 ± 4.3	2.5 ± 4.2
Polyunsaturated fatty acids (% of total energy intake)	≤ 2	≥ 10	5.1 ± 3.2	4.9 ± 3.1
Sodium (mg/day)[j]	highest decile	lowest decile	4.9 ± 3.2	5.1 ± 3.1
Alcohol (drinks/day)[k]			4.6 ± 3.4	4.4 ± 3.8
Women	≥ 2.5	0.5 - 1.5		
Men	≥ 3.5	0.5 - 2.0		
**Total**	**0**	**110**	49.6 ± 14.3	47.8 ± 14.3

Adapted from Chiuve et al (19)
[a] Intermediate food intake was scored proportionately between the minimum score 0 and the maximum score 10.
[b] One serving was equal to 118.3g of raw or cooked vegetables, 30g of dried vegetables or 250g of homemade vegeTable S oup. All vegetables, leafy vegetables, sprouts, green beans, peas, sweat corn, root vegetables, cabbages, avocadoes, mushrooms, onions, seaweeds, homemade vegeTable S oups. Except: potatoes products, olives, herbs, vegetables juices.
[c] One serving was equal to 118.3g of raw or cooked fruit or 30g of dried fruit. All fruits, except fruit juices, fruit jams, and candied fruit.
[d] All bread products, flours, breakfast cereals, cereal flakes and brans, dough, pasta, rice, spätzle, other cereal grains (e.g., quinoa, barley) with a carbohydrate-to-fiber ratio smaller than 10:1.
[e] One serving was equal to 226.8g. Sweetened soft drinks, sports and energy drinks, fizzy drinks, diluted syrup, ice tea, alcoholic drinks substitutes, drinks made with fruit juices (e.g. lemonades, nectars), 100% fruit juices, smoothies. Except: drinks with artificial sweeteners (e.g. light or sugar-free soft drinks).
[f] One serving was equal to 28.4g. Nuts, seeds, legumes, meat substitutes, soy products.
[g] One serving was equal to 113.4g of red meat or 42.5g of processed meat. Fresh meat of mammals, offal, wild meat, sausages, cold cuts, smoked and cured meat.
[h] According to the Swiss regulation, each food item must contain max. 2g of trans fat per 100g of total fat.
[i] Fish, seafood. Except: processed fish (e.g., fish in crumbs), seafood products (e.g., surimi).
[j] Values in highest decile were ≥ 3963 mg/d in women and ≥5672 mg/d in men and in lowest decile : ≤1361 mg/d in women and ≤1889 mg/d in men.
[k] One drink was 113.4g of wine, 340.2g of beer or 42.5g of liquor/spirit. A score of 2.5 was given to non-drinkers.

Table S3. Mediterranean diet score (MDS) components and scoring, menuCH, Switzerland, 2014-2015

**Components**	**Food items included**	**Criteria for minimum score (0)**	**Criteria for maximum score (1)**
Vegetables (g/day)	All vegetables, leafy vegetables, sprouts, green beans, peas, sweat corn, root vegetables, cabbages, avocadoes, mushrooms, onions, seaweeds Except: potatoes products, olives, herbs, vegetables juices, vegeTable S	< median	> median
Legumes (g/day)	All legumes (weight after cooking)	< median	> median
Fruits and nuts (g/day)	All fruits, nuts, seeds Except: fruit juices, fruit jams, candied fruit	< median	> median
Cereals (g/day)	Bread, bread products (e.g. croissants, focaccia), crisp bread, flours, starches, natural cereal flakes, breakfast cereals, pasta, rice, spätzle, other cereal grains (e.g., quinoa, barley), dough, pastry	< median	> median
Fish (g/day)	Fish, seafood, processed fish and seafood products	< median	> median
Meat (g/day)	Red meat (e.g., beef, porc, lamb, venison), poultry, processed meat (e.g., sausages, cold cuts, smoked and cured meat), offals	> median	< median
Dairy products (g/day)	Milk, fermented milk, milk-based drinks (e.g. hot chocolate, milk in coffee mixes), yogurt, kefir, fresh cheese, spread cheese, soft cheese, hard cheese, cream, desserts made with dairy products (e.g. caramel cream), milk-based ice creams. Except: vegetal dairy substitutes (e.g. soya milk)	> median	< median
Alcohol (g/day)	Beer, wine, champagne, wine products, port, sherry, vermouth, cocktails, liquors, spirits, long drinks		
Women		< 5 g/day or > 25 g/day	5-25 g/day
Men		< 10 g/day or > 50 g/day	10-50 g/day
Ratio of monounsaturated to saturated fatty acids		< median	> median
Total		**0**	**9**

Adapted from Trichopoulou et al. 2003 and Pestoni et al. 2018

Table S4. Description of included sample, menuCH, Switzerland, 2014-2015

				Educational level, men only			Educational level, women		
	Men	Women	*p*	Tertiary	Secondary/primar	*p*	Tertiary	Secondary/primar	*p*
N	851	1009		503	348		469	540	
Age, mean (SD)	50.1 (14.0)	48.5 (14.1)	*0.02*	49.3 (13.9)	51.2 (14.2)	*0.01*	44.1 (13.6)	52.4 (13.5)	*<0.001*
AHEI, mean (SD)	46.5 (14.5)	50.7 (13.9)	*<0.001*	48.2 (14.9)	45.0 (13.6)	*0.01*	52.2 (13.5)	49.4 (14.4)	*0.03*
Obese, n (%) by marker									
Body mass index	103 (12.1)	93 (9.2)	*0.04*	41 (8.2)	62 (17.9)	*<0.001*	29 (6.2)	64 (11.9)	*0.01*
Waist circumference	162 (19.1)	182 (18.5)	*0.71*	80 (15.9)	82 (23.8)	*<0.01*	48 (10.6)	134 (25.1)	*<0.001*
Waist-to-hip ratio	407 (48.1)	142 (14.4)	*<0.01*	217 (43.1)	190 (55.2)	*0.001*	35 (7.7)	107 (20.1)	*<0.001*
Waist-to-height ratio	489 (57.8)	298 (30.2)	*<0.01*	262 (52.1)	227 (66.2)		82 (18.1)	216 (40.5)	*<0.001*

AHEI, Alternate Healthy Eating Index. Statistical differences between educational groups for categorical variables assessed by Chi-square test, for age and the AHEI using student T-test.

Table S5. Association of educational level with obesity outcomes, and the mediation of diet (AHEI) in this association, assessed via the difference method, menuCH, Switzerland, 2014-2015

	Model 1	Model 1 + AHEI	
Obesity marker	OR (95% CI)	OR (95% CI)	% attenuated
Body mass index	2.96 (1.91, 4.59)	2.60 (1.67, 4.06)	12.0
Waist circumference	2.64 (1.86, 3.75)	2.35 (1.65, 3.35)	12.0
Waist-to-hip ratio	2.71 (1.89, 3.88)	2.39 (1.66, 3.44)	12.6
Waist-to-height ratio	3.01 (2.16, 4.19)	2.62 (1.88, 3.67)	12.6

AHEI, Alternate healthy eating index; OR, odds ratio; CI, confidence interval. Odds ratios and 95% confidence intervals, adjusted for age, sex, physical activity, and total energy intake using logistic regression models. AHEI, in quintiles, as categorical variable. % attenuated = [(coeff of model 1) – (coeff of model 1 + AHEI)]/(coeff of model 1) * 100.

Table S6. Association between educational level[a] and quintiles of the Alternate Healthy Eating Index, menuCH, Switzerland, 2014-2015

		Middle vs higher education	Lower vs higher education
AHEI quintiles	Mean (SD)	OR (95% CI)	OR (95% CI)
Healthiest	69.5 (7.1)	1.00 (reference)	1.00 (reference)
Healthier	55.6 (2.8)	0.78 (0.56, 1.11)	1.05 (0.73, 1.52)
Middle	47.2 (2.3)	1.17 (0.82, 1.65)	1.40 (0.96, 2.02)
Unhealthier	39.5 (2.3)	1.25 (0.87, 1.65)	2.00 (1.37, 2.90)
Unhealthiest	29.1 (4.6)	1.47 (1.02, 2.18)	2.88 (1.94, 4.29)
p-trend [a]		*<0.001*	*<0.001*

AHEI, Alternate Healthy Eating Index; OR, odds ratio; CI, confidence interval. Odds ratio and 95% confidence interval adjusted for age, sex and physical activity, from ordered logistic regression, comparing likelihood of being in each quintile of the AHEI for middle (higher secondary education) versus higher (tertiary education), and for lower (lower secondary or primary education) versus higher education.

[a] Educational level categorized as 1) tertiary, 2) higher secondary, and 3) lower secondary or primary education.

[a] P for trend from linear regression with education as continuous variable

Table S7. Results from counterfactual mediation of diet quality (AHEI) in the association of educational level[a] with obesity markers, menuCH, Switzerland, 2014-2015

	MTE	NDE	NIE	PM
Obesity marker	OR (95% CI)	OR (95% CI)	OR (95% CI)	% (95% CI)
Body mass index	5.05 (2.75, 11.01)	4.04 (2.11, 8.90)	1.25 (1.07, 1.58)	25.0 (8.5, 47.3)
Waist circumference	3.59 (2.25, 6.33)	2.71 (1.64, 4.76)	1.33 (1.14, 1.60)	34.0 (16.5, 55.0)
Waist-to-hip ratio	3.46 (2.32, 5.43)	2.57 (1.75, 3.89)	1.35 (1.17, 1.63)	36.4 (21.0, 55.9)
Waist-to-height ratio	3.77 (2.28, 7.05)	3.06 (1.75, 5.93)	1.23 (1.06, 1.47)	25.5 (8.6, 46.1)

AHEI, Alternate Healthy Eating Index; MTE, marginal total effects; NDE, natural direct effect; NIE, natural indirect effect; OR, odds ratio; CI, confidence interval. Odds ratio and 95% confidence interval for the total effect of the exposure on the outcome (MTE), for the effect of the exposure on the outcome via pathways that exclude the mediator (NDE); the effect of the exposure on the outcome via the mediator (NIE). PM, proportion of the association between educational level and obesity markers which is mediated by diet quality, estimated using the AHEI, adjusted for age, sex, physical activity, total energy intake, and smoking behavior.

[a] Educational level categorized as 1) tertiary, 2) higher secondary, and 3) lower secondary or primary education.

Table S8. Results from counterfactual mediation of diet quality (MDS) in the association of educational level with obesity markers, menuCH, Switzerland, 2014-2015

	MTE	NDE	NIE	PM
Obesity marker	OR (95% CI)	OR (95% CI)	OR (95% CI)	% (95% CI)
Body mass index	2.25 (1.59, 3.38)	2.14 (1.52, 3.18)	1.05 (1.01, 1.11)	8.9 (2.0, 18.6)
Waist circumference	1.70 (1.29, 2.33)	1.62 (1.23, 2.20)	1.05 (1.02, 1.10)	12.4 (4.1, 26.5)
Waist-to-hip ratio	1.70 (1.28, 2.30)	1.61 (1.23, 2.18)	1.06 (1.02, 1.11)	13.0 (4.4, 28.2)
Waist-to-height ratio	1.88 (1.47, 2.46)	1.76 (1.39, 2.30)	1.07 (1.03, 1.12)	13.5 (6.1, 23.5)

MDS, Mediterranean Diet Score; MTE, marginal total effects; NDE, natural direct effect; NIE, natural indirect effect; OR, odds ratio; CI, confidence interval. Odds ratio and 95% confidence interval for the total effect of the exposure on the outcome (MTE), for the effect of the exposure on the outcome via pathways that exclude the mediator (NDE); the effect of the exposure on the outcome via the mediator (NIE). PM, proportion of the association between educational level and obesity markers which is mediated by diet quality, estimated using the MDS, adjusted for age, sex, physical activity, total energy intake, and smoking behavior.

Table S9. Association between income level and quintiles of the Alternate Healthy Eating Index, menuCH, Switzerland, 2014-2015

		Middle vs higher income	Lower vs higher income
AHEI quintiles	Mean (SD)	OR (95% CI)	OR (95% CI)
Healthiest	69.4 (7.1)	1.00 (reference)	1.00 (reference)
Healthier	55.6 (2.8)	1.04 (0.72, 1.51)	1.22 (0.85, 1.74)
Middle	47.2 (2.3)	1.43 (0.99, 2.05)	1.18 (0.82, 1.70)
Unhealthier	39.4 (2.4)	1.75 (1.21, 2.54)	1.53 (1.05, 2.22)
Unhealthiest	29.1 (4.7)	1.54 (1.04, 2.26)	1.54 (1.05, 2.25)
p-trend [a]		*<0.01*	*<0.01*

AHEI, Alternate Healthy Eating Index; OR, odds ratio; CI, confidence interval. Odds ratio and 95% confidence interval adjusted for age, sex and physical activity, from ordered logistic regression, comparing likelihood of being in each quintile of the AHEI for middle vs high income and low vs high income.

[a] P-value for linear trend

.

Table S10. Association between quintiles of the Alternate Healthy Eating Index and obesity markers, menuCH, Switzerland, 2014-2015

	AHEI quintiles					
	Healthiest	Healthier	Middle	Unhealthier	Unhealthiest	
Obesity marker	Reference	OR (95% CI)	OR (95% CI)	OR (95% CI)	OR (95% CI)	P-trend[a]
Body mass index	1.00	1.26 (0.71, 2.24)	1.87 (1.09, 3.19)	1.87 (1.09, 3.22)	3.32 (1.96, 5.60)	*<0.0001*
Waist circumference	1.00	1.48 (0.96, 2.27)	1.66 (1.09, 2.54)	2.01 (1.31, 3.07)	3.07 (2.00, 4.71)	*<0.0001*
Waist-to-hip ratio	1.00	1.67 (1.12, 2.50)	1.88 (1.26, 2.81)	2.52 (1.69, 3.78)	3.19 (2.12, 4.81)	*<0.0001*
Waist-to-height ratio	1.00	1.72 (1.22, 2.43)	2.01 (1.42, 2.84)	2.75 (1.93, 3.91)	3.43 (2.38, 4.93)	*<0.0001*

AHEI, Alternate Healthy Eating Index. Odds ratio and 95% confidence interval for the likelihood of being in obesity category according to each obesity marker, for individuals in each quintile of the AHEI *relative to those in the highest (healthiest) quintile* (reference group), adjusted for age, sex, physical activity, and total energy intake.

[a] Trend across quintiles of AHEI.

Table S11. Counterfactual mediation of diet quality (AHEI) in the association of income level with obesity markers, menuCH, Switzerland, 2014-2015

	MTE	NDE	NIE	PM
Obesity marker	OR (95% CI)	OR (95% CI)	OR (95% CI)	% (95% CI)
Body mass index	2.12 (1.11, 4.13)	1.90 (1.02, 3.65)	1.12 (1.01, 1.31)	19.8 (-1.3, 73.6)
Waist circumference	1.93 (1.16, 3.39)	1.75 (1.06, 3.06)	1.10 (1.00, 1.26)	18.7 (-0.1, 58.0)
Waist-to-hip ratio	1.86 (1.13, 3.20)	1.63 (1.01, 2.76)	1.14 (1.01, 1.33)	26.5 (-1.0, 81.7)
Waist-to-height ratio	1.55 (0.96, 2.47)	1.37 (0.87, 2.14)	1.13 (1.01, 1.30)	32.6 (-53.4, 160.2)

AHEI, Alternate Healthy Eating Index; MTE, marginal total effects; NDE, natural direct effect; NIE, natural indirect effect. Odds ratio and 95% confidence interval for the total effect of the exposure on the outcome (MTE), for the effect of the exposure on the outcome via pathways that exclude the mediator (NDE); the effect of the exposure on the outcome via the mediator (NIE). PM, proportion of the association between income level and obesity markers which is mediated by diet quality, estimated using the AHEI, adjusted for age, sex, physical activity, total energy intake, and smoking behavior.

Figure S1. Flowchart of participant inclusion, menuCH, Switzerland, 2014-2015

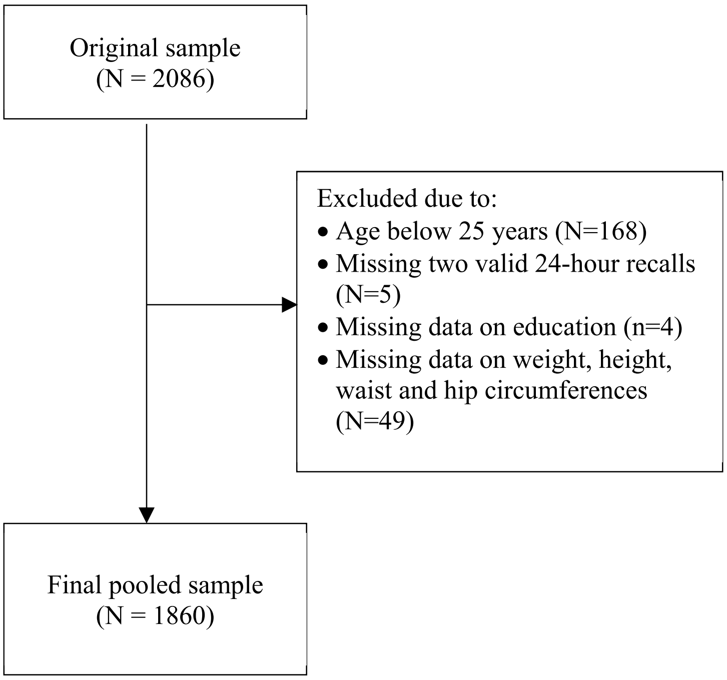

Supplement: Supplementary file 1 [file nutrients-11-01573-s001.pdf]
